# Dietary Customs and Social Deprivation in an Aging Population From Southern Italy: A Machine Learning Approach

**DOI:** 10.3389/fnut.2022.811076

**Published:** 2022-03-07

**Authors:** Rossella Tatoli, Luisa Lampignano, Rossella Donghia, Fabio Castellana, Roberta Zupo, Ilaria Bortone, Sara De Nucci, Giuseppe Campanile, Domenico Lofù, Luigi Vimercati, Madia Lozupone, Giovanni De Pergola, Francesco Panza, Gianluigi Giannelli, Tommaso Di Noia, Heiner Boeing, Rodolfo Sardone

**Affiliations:** ^1^National Institute of Gastroenterology—IRCCS “Saverio de Bellis”, Castellana Grotte, Italy; ^2^Department of Electrical and Information Engineering, Polytechnic of Bari, Bari, Italy; ^3^Interdisciplinary Department of Medicine, Section of Occupational Medicine B. Ramazzini, School of Medicine, University of Bari Aldo Moro, Bari, Italy; ^4^Department of Basic Medicine, Neuroscience, and Sense Organs, University of Bari Aldo Moro, Bari, Italy; ^5^Unit of Internal Medicine and Geriatrics, National Institute of Gastroenterology “S. de Bellis” Research Hospital, Castellana Grotte, Italy; ^6^Department of Biomedical Science and Human Oncology, School of Medicine, University of Bari Aldo Moro, Bari, Italy; ^7^Department of Epidemiology, German Institute of Human Nutrition Potsdam-Rehbruecke, Nuthetal, Germany

**Keywords:** social deprivation, older, diet, population study, dietary pattern

## Abstract

**Background:**

Diet and social determinants influence the state of human health. In older adults, the presence of social, physical and psychological barriers increases the probability of deprivation. This study investigated the relationship between social deprivation and eating habits in non-institutionalized older adults from Southern Italy, and identified foods and dietary habits associated with social deprivation.

**Methods:**

We recruited 1,002 subjects, mean age 74 years, from the large population based Salus in Apulia Study. In this cross-sectional study, eating habits and the level of deprivation were assessed with FFQ and DiPCare-Q, respectively.

**Results:**

Deprived subjects (*n* = 441) included slightly more females, who were slightly older and with a lower level of education. They consumed less fish (23 vs. 26 g), fruiting vegetables (87 vs. 102 g), nuts (6 vs. 9 g) and less “ready to eat” dishes (29 vs. 33 g). A Random Forest (RF) model was used to identify a dietary pattern associated with social deprivation. This pattern included an increased consumption of low-fat dairy products and white meat, and a decreased consumption of wine, leafy vegetables, seafood/shellfish, processed meat, red meat, dairy products, and eggs.

**Conclusion:**

The present study showed that social factors also define diet and eating habits. Subjects with higher levels of deprivation consume cheaper and more readily available food.

## Introduction

Together with the quality of diet and eating habits, social determinants influence the welfare of a population (1–3). An unhealthy social environment can adversely affect an individual's state of health, increasing the risk of several pathological conditions (4). The more disadvantaged groups suffer from higher rates of obesity (5, 6), diabetes (7), cardiovascular disease, osteoporosis (8), dental caries (9), and some forms of cancer (10). All of these diseases have a direct link to nutrition and diet (11, 12). Social and economic factors exert a strong influence on food choices and on the possibility of accessing food, helping to explain some evident inequalities in terms of health status. In fact, morbidity and mortality rates in industrialized societies follow a socioeconomic gradient (13–15).

Wrezinski defines deprivation as “the lack of safety, like a job, enabling individuals and families to assume professional, family and social responsibilities and to enjoy basic rights” (16). In the same year, Townsend identified “social deprivation” as one of the leading causes of health inequalities, defining it as a “state of observable and demonstrable disadvantage relative to the local community or the wider society to which an individual, family or group belongs” (17). In his definition of deprivation, Townsend shifted the focus to conditions rather than resources, introducing a difference between deprivation and poverty, and identifying deprivation as the leading cause of inequalities in health (17, 18).

The presence of social, physical and psychological barriers, such as loss of appetite, an impaired sense of smell and taste, difficulty in finding and preparing food, age-related diseases, reduced mobility, and social isolation, not only make states of deprivation more frequent in older adults than in younger ones, but also result in inevitable changes of lifestyle and eating habits. Many age-related physical, clinical, economic and social factors can compromise previously existing healthy dietary habits. In qualitative and quantitative terms, several studies have highlighted that nutrition in older people is influenced by age, gender, and socio-economic conditions (19, 20). In this age group, good nutrition is a fundamental factor in preserving good cognitive and functional abilities, and more generally, in guaranteeing an optimal state of health (21). Older people are known to be at greater risk for nutritional disorders and malnutrition, including undernutrition, and nutrient deficiencies and imbalances (22).

According to the data reported by the National Institute of Statistics (ISTAT), about 22.8% of the entire Italian population is aged over 65 years of age, and the aging population trend is continually rising (www.istat.it).

Despite the solid demographic weight of this age group in the Italian territory and the close link between diet, eating habits, and psycho-socio-economic conditions, in the scientific literature there are currently no studies evaluating these aspects in this older Italian population. Even of non-Italian older adult populations, few studies have been made. Some have evaluated deprivation but with a different focus. For example, Holmes and Roberts investigated the influence of social and physical factors on diet quality but in materially deprived (low income) older people of the United Kingdom (23). The present study aimed to investigate the relationship between social deprivation and eating habits among non-institutionalized older adults from Southern Italy, using a machine learning approach. This particular technique can exclude the effect of correlation of particular foods, in order to extrapolate the prediction power of Social Deprivation and explore whether social deprivation is predictive of a certain dietary pattern.

## Materials and Methods

### Study Design and Population

This cross-sectional population-based study involved 1,002 subjects aged over 64 years, representing a sub-sample from the “Salus in Apulia Study” (*n* = 4,537), enrolled from 2014 to 2019 in Castellana Grotte, Southern Italy. The sample is representative of the entire population of older people (age > 65 years) of Castellana Grotte at 2014, as described elsewhere (24). Salus in Apulia is a public health initiative funded by the Italian Ministry of Health and Apulia Regional Government and conducted at IRCCS “S. de Bellis” Research Hospital in Castellana Grotte. This selection of study participants allowed us to utilize past individual data, generated from other investigations (24). For the present study, subjects with nutritional and social assessments were selected. All participants signed an informed consent form before their examination. The IRB of the head institution, the National Institute of Gastroenterology and Research Hospital “S. de Bellis” in Castellana Grotte, Italy, approved the study. The study was conducted in accordance with the Helsinki Declaration of 1975 and adhered to the “Standards for Reporting Diagnostic Accuracy Studies” (STARD) guidelines (http://www.stard-statement.org/). The manuscript was organized according to the “Strengthening the Reporting of Observational Studies in Epidemiology-Nutritional Epidemiology” (STROBE-nut) guidelines (https://www.strobe-nut.org/).

### Social Deprivation, Lifestyle, and Clinical Assessment

Social deprivation was assessed with the Deprivation in Primary Care Questionnaire (DiPCare-Q). The signs of deprivation, perceived and self-reported, allow possible states of social distress to be identified. DiPCare-Q is a structured self-administered questionnaire consisting of 16 questions about social status, level of education, source of income, and poverty level. This questionnaire assesses three distinct dimensions of deprivation: material (eight items), social (five items), and health (three items), each of which corresponds to a sub-index (25). Sub-indexes for material, social and health deprivation were calculated by adding one point for each positive answer. Social deprivation and health indexes could be assumed to be linearly correlated to subjective social status, whereas material deprivation could not. The score value attributed to each participant is a direct function of the score: the higher the value of the index, the more deprived the subject. Since social deprivation depends on the socio-economic status of each peculiar population, we considered as socially deprived those subjects with a DiPCare-Q score greater than the median value (1) for the Salus in Apulia sub-population.

Global cognitive performance was assessed by the Mini-Mental State Examination (MMSE), a measure that includes 10 items determining spatial and temporal orientation, attention, memory, language, and visuospatial functions (26).

Lifestyle and anthropometric assessments were evaluated by a physician during an interview at the study center. Smoking status was assessed with the single question, “Are you a current smoker?.” The level of education was expressed as years of schooling. Body mass index (BMI) was calculated as kg/m^2^. Height and weight measurements were performed using a Seca 220 stadiometer and a Seca 711 scale. Blood was collected from the subjects in the morning after an overnight fast and, among other parameters, Interleukin-6 (IL-6), Tumor Growth Factor-α(TNF-α), C-Reactive Protein (CRP) were measured, using standard automated enzymatic colorimetric methods (AutoMate 2550, Beckmann Coulter, Brea, Ca, US), under strict quality control.

### Dietary Assessment

Diet and eating habits were assessed with a validated food frequency questionnaire (FFQ) used in previous studies (27, 28). The self-administered questionnaire was checked for completeness by a registered dietitian during an interview at the study center. For these foods, the frequency of intake of a predefined portion over the last year was probed in the questionnaire via a scale of 9 categories. Each portion was given a weight expressed in grams. The self-administered FFQ was initially structured in 11 sections listing foods of similar characteristics: grains, meat, fish, milk and dairy products, vegetables, legumes, fruits, miscellaneous foods, water and alcoholic beverages, olive oil and other edible fats, coffee/sugar and salt. The FFQ was then validated against dietary records, and the results were reviewed to adapt the questionnaire to our population (29). In the final questionnaire, 85 food items were considered to best reflect the regional diet, together with some questions about the use of edible fats. The 85 food items in the FFQ and the questions about the use of fat have been regrouped for statistical analyses and further summarized under 28 food groups (24). One food group (edible cooking fats) could not be quantified and was not used in the present study. The FFQ referred only to the frequency of intake and did not consider differences in portion sizes.

### Statistical Analysis

Subject characteristics are reported as Mean ± Standard Deviation (M ± SD) for continuous variables and as frequencies and percentages (%) for categorical variables.

Comparisons of a categorical variable between two independent groups were performed by Chi-square test and Wilcoxon rank-sum (Mann-Whitney) test to assess the statistically significant differences between groups in relation to a continuous variable based on mean rank scores.

A logistic regression model was used to evaluate the associations of Social Deprivation with age, gender, education and the single Food-Group examined and to obtain the z-score to evaluate the ratio of the coefficient estimate divided by standard error of the estimator, i.e., the point estimate of the coefficient, with 95% Confidence Intervals (95% C.I.). The Random Forest (RF) method (30), is a machine supervised learning algorithm based on a randomized decisional tree, for ranking the prediction power of a set of variables regarding the outcome of interest (Supplementary Table 1).

The “forest” it builds is an ensemble of decision trees, usually trained with the “bagging” method. The general idea of the bagging method is that a combination of learning models increases the overall result.

We examined which variables best predict variance in the intervention effects by ranking the covariates in order of importance. The ranking is calculated as the sum of how often a given covariate is split at each depth of the forest. The sum is weighted so that early splits (low forest depth) are more important than late splits. Variables are considered “more important” if the variable is more frequently used for the first splits across all decision trees that are grown in the random forest.

The parameter used for ranking was the importance score variable, calculated by adding up the improvement in the objective function given by the splitting criterion over all internal nodes of a tree and across all trees in the forest, separately for each predictor variable. The importance score variable was normalized by dividing all scores over the maximum score (100%).

Variables with a high importance (age, gender, education, and 28 food groups) were drivers of the outcome and their score values have a significant impact on the outcome, in this case social deprivation. When testing the null hypothesis of no association, the probability level of error at two tails was set at 0.05. All statistical computations were made using StataCorp. 2021. Stata Statistical Software: Release 17. College Station, TX: StataCorp LLC.

## Results

In the participants examined in the present study (*n* = 1,002), belonging to the “Salus in Apulia Study” population, the female sex was slightly predominant, accounting for 51%.

The average age of the sample was 74 years, with a standard deviation of 6.47, a median age of 73.04 and an interquartile range of 68.75-78.79 years. The study population was subdivided into two groups based on the median value of the deprivation index. The group of “less deprived” (LD) subjects included people with a deprivation index value of 0 and 1, while the group of “more deprived” (MD) people had a deprivation index value ranging between 2 and 5.

Table 1 shows differences between the two groups for some of the parameters analyzed. Among the 1,002 participants of the present study, 441 were classified as more deprived. MD participants included more females, were slightly older, and had a lower educational level (mean 6.8 years). In their blood, higher Inflammation biomarkers could be detected (IL-6: 3.61 pg/ml ± 5.91 vs. 4.78 pg/ml ± 8.85, *p*: 0.01; TNF-α: 2.84 μg/ml ± 4.08 vs. 3.04 μg/ml ± 4.29, *p*: 0.40; CRP: 0.54 mg/dl ± 0.71 vs. 0.60 mg/dl ± 0.90, *p*: 0.30). They had lower scores of MMSE than LD participants (25.76 ± 4.54 vs. 27.33 ± 3.38, *p*: < 0.0001).

**Table 1:** Sociodemographic and clinical variables of the study population categorized according to the Deprivation in the Primary Care Questionnaire (DiPCare-Q) score.

Parameters^*^	**DipCare-Q score**		
	**0-1 (*n =* 561)**	**2-5 (*n =* 441)**	***P* ^ψ^**
Sex (%)			0.04^§^
M	291 (51.87)	200 (45.35)	
F	270 (48.13)	241 (54.65)	
Age (years)	73.11 ± 6.29	75.33 ± 6.48	<0.0001
Age classes (%)			<0.001^§^
65-70	228 (40.64)	103 (23.36)	
70-75	129 (22.99)	127 (28.80)	
75-80	111 (19.79)	97 (22.00)	
80-85	62 (11.05)	79 (17.91)	
85-90	26 (4.63)	29 (6.58)	
>90	5 (0.89)	6 (1.36)	
Education (years)	7.74 ± 3.92	5.89 ± 3.42	<0.0001
Smoking (Yes) (%)	45 (8.02)	34 (7.71)	0.86^§^
BMI (Kg/m^2^)	28.07 ± 4.71	28.75 ± 5.21	0.10
			
IL-6 (pg/ml)	3.61 ± 5.91	4.78 ± 8.85	0.01
TNF-α (μg/ml)	2.84 ± 4.08	3.04 ± 4.29	0.40
CRP (mg/dl)	0.54 ± 0.71	0.60 ± 0.90	0.30

*The Salus in Apulia Study (n = 1,002)*.

* *As Mean and Standard Deviation for continuous, and percentage (%) for categorical variables*.

*BMI, Body Mass Index; IL-6, Interleukin-6; TNF-α, Tumor Growth Factor-α; CRP, C-Reactive Protein*.

ψ *Wilcoxon rank-sum test (Mann-Whitney)*,

§ *Chi-Square or Fisher's test, as appropriate*.

Table 2 shows the characteristics of the two groups in terms of food group consumption. MD subjects consumed less fish (23.4 ± 22.4 g vs. 26.4 ± 23.5 g, *p*: 0.01), fruiting vegetables (87.3 ± 70.2 g vs. 101.5 ± 92.4 g, *p*: 0.04), nuts (5.8 ± 13.9 g vs. 8.8 ± 17.6 g, *p*: 0.004) and less “ready to eat” dishes (28.7 ± 31.7 g vs. 32.6 ± 32.4 g, *p*: 0.01), compared to LD subjects.

**Table 2:** Food groups average consumption (expressed in grams) categorized according to the Deprivation in the Primary Care Questionnaire (DiPCare-Q) score.

Parameters^*^	**DipCare-Q score**		
	**0-1 (*n* = 561)**	**2-5 (*n* = 441)**	***P* ^ψ^**
**Food groups^¥^**			
Dairy	112.1 ± 116.1	100.4 ± 101.7	0.15
Low fat dairy	98.6 ± 108.1	102.3 ± 110.1	0.94
Eggs	8.4 ± 8.9	7.6 ± 7.9	0.32
White meat	25.9 ± 33.5	26.2 ± 33.4	0.85
Red meat	23.9 ± 30	22 ± 16.2	0.94
Processed meat	15.4 ± 15.9	14.1 ± 13.3	0.38
Fish	26.4 ± 23.5	23.4 ± 22.4	0.01
Seafood/shellfish	9.3 ± 9.4	9 ± 10.8	0.14
Leafy vegetables	62.3 ± 71.7	55.8 ± 62.3	0.16
Fruiting vegetables	101.5 ± 92.4	87.3 ± 70.2	0.04
Root vegetables	12.2 ± 26.9	11.5 ± 24.3	0.17
Other vegetables	85.6 ± 89.6	74.2 ± 67.2	0.17
Legumes	38.4 ± 34.7	37.8 ± 29.2	0.35
Potatoes	13.6 ± 22.8	13.6 ± 16.6	0.29
Fruits	605.9 ± 522.8	619.9 ± 541.2	0.73
Nuts	8.8 ± 17.6	5.8 ± 13.9	0.004
Grains	153.2 ± 104.6	157.1 ± 109.7	0.50
Olives and vegetable oil	54.2 ± 46.3	51.9 ± 34.5	0.48
Sweets	24.1 ± 36.6	23.6 ± 40.6	0.45
Sugary food	10.7 ± 20.9	11.2 ± 27.2	0.57
Juices	6.3 ± 19.9	6.8 ± 18.7	0.87
Caloric drinks	5.8 ± 35.3	10.7 ± 66.1	0.36
Ready to eat dishes	32.6 ± 32.4	28.7 ± 31.7	0.01
Coffee	47.7 ± 31.4	43.6 ± 27.8	0.10
Wine	120.8 ± 160.4	115.3 ± 165.5	0.21
Beer	14.6 ± 64.0	18.6 ± 74.6	0.46
Spirits	1.3 ± 4.2	1.4 ± 6.4	0.15
Water	657.9 ± 297.0	652.6 ± 303.1	0.76

*The Salus in Apulia Study (n = 1,002)*.

* *Mean and Standard Deviation for continuous, and percentage (%) for categorical variables.*

ψ *Wilcoxon rank-sum test (Mann-Whitney)*.

¥ *Food Groups and Nutrients were calculated on quantity of daily consumption*.

**Figure 1:**
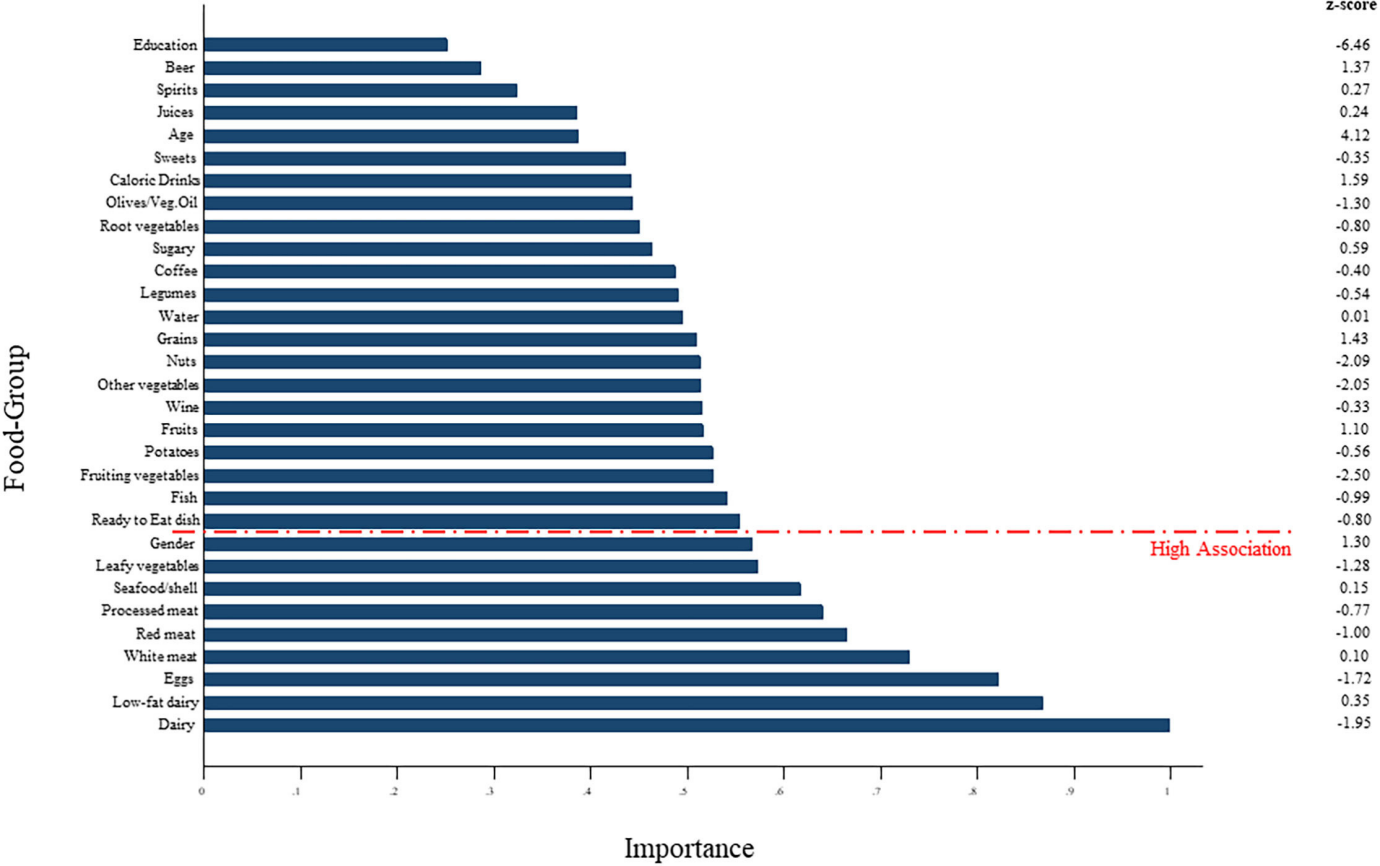
Random Forest (RF) model used to identify a dietary pattern associated with social deprivation.

A Random Forest (RF) model was used to identify a dietary pattern associated with social deprivation. This analysis underlined that 8 of the 28 food groups (with an importance value exceeding 0.50) were associated with this outcome: leafy vegetables, seafood/shellfish, processed and red meat, eggs, white meat, dairy and low-fat dairy products (Figure 1). In particular, the model showed that low-fat dairy products (z-score: 0.35), seafood/shellfish (z-score: 0.15), and white meat (z-score: 0.1) were positively associated with social deprivation. By contrast, leafy vegetables (z-score: −1.28), dairy products (z-score: −1.95), processed meat (z-score: −0.77), red meat (z-score: −1.00), and eggs (z-score: −1.72), were negatively associated with deprivation.

## Discussion

In our study of 1,002 older adults, we identified some foods able to rank subjects according to social deprivation. A higher consumption of low-fat dairy products, white meat and seafood/shellfish and a lower consumption of leafy vegetables, dairy products, processed meat, red meat and eggs was associated with a higher level of social deprivation.

Age was one of the factors most significantly associated with social deprivation. Older adults are generally more deprived than young subjects due to the various barriers, social and otherwise, that typically affect this age group. In our study sample, the average age of the more socially deprived (MD) group was 75 years while less socially deprived (LD) subjects were slightly younger, with an average age of 73 years, supporting the concept that age is positively associated with social deprivation. The level of education also contributes to define a more or less deprived subject. Although we could not identify any study that directly assessed the relationship between social deprivation and level of education, Prattley et al. found that fewer years of schooling predict higher levels of social exclusion in later life (31). Education and awareness of health issues and socioeconomic status impact the diet quality: wealthier, and more educated older people have a better diet (32). One of the indicators of diet quality is the consumption of fruits and vegetables. Shohaimi et al. showed that social class, educational level, and deprivation level of the residential area independently predict fruit and vegetable consumption: people in manual labor social classes, with no educational qualifications, or those who live in the most deprived areas, consumed significantly lower amounts of fruit and vegetables compared to those in non-manual labor social classes (33). In Italy, the differences in this indicator by level of education are well established, in all age groups, highlighting a notable advantage for more highly educated men and women (https://www.istat.it/it/files//2021/03/BES_2020.pdf accessed September 2021).

It was of great interest to note that the prediction power of education level in our study population is not as important as that of diet for social deprivation, although our study sub-sample had an average of only about 7 years of school (34). This result leads us to hypothesize that other factors, including dietary ones, have a greater importance in defining the level of deprivation. The most important finding in our study was the profiling of a dietary pattern associated with social deprivation. In this sense, the creation of food groups starting from single food items was very useful. In the MD subjects, the consumption of low-fat dairy and white meat was higher than in the LD subjects, probably because these are cheap foods and easily available. By contrast, they ate less of the other foods in this category, which are generally more expensive and more difficult to find. Moreover, these foods, in particular wine, dairy, processed and red meat, are typically eaten at convivial meals such as parties and banquets. This could further explain the background of social deprivation. It is interesting that in another study in this area, fatty meats and eggs were associated with a lower risk of mortality (35). At that time, we discussed the finding in relation to the provision of dietary energy, that was always a problem in the past among hard working agricultural professions. Now, we could add the economic and social side of deprivation to the discussion.

In our sample, in the light of the data shown in Table 2 and the ranking (Figure 1) we might hypothesize that the diet of MD subjects was poor of fish, nuts, fresh fruits and vegetables, that are rich of some micronutrients and phytochemicals absent in meat, eggs and dairy products. For this reason, the diet of MD older subjects could be nutritionally unbalanced. Moreover, our study showed that LD subjects ate more fish (Table 2), an important source of polyunsaturated lipids, in particular Ω-3, nuts, an important source of monounsaturated lipids, in particular oleic acid, vitamins and minerals, including magnesium and selenium, and vegetables, in particular fruiting vegetables, that are important sources of phytochemicals (www.bda-ieo.it). To confirm this data, longitudinal data are needed.

The concept of deprivation is vast; social deprivation is currently distinguished from material deprivation, even if the two are really intimately linked: people are considered deprived if they lack the material standards of diet, clothing, housing, household facilities, working, environmental and locational conditions and facilities which are ordinarily available in their society, and do not participate in or have access to the forms of employment, occupation, education, recreation and family and social activities and relationships which are commonly enjoyed or accepted (17). The achievement of specific dietary standards, understood in terms of quantity and quality of foods eaten, can be considered to be almost inevitably influenced by socioeconomic factors. It is now acknowledged that diet quality is very much a function of socioeconomic status: the energy cost of fresh produce is 10-fold that of vegetable oils and sugars, and the difference in energy costs between healthy and unhealthy foods is several thousand per cent (32). A higher consumption of fats and sweets was shown to be associated with a net saving in dietary costs: replacing fats and sweets with more vegetables and fruits was associated with an increase in expenses (36). The influence of socioeconomic aspects on dietary standards might explain a greater consumption of ready to eat dishes and meats, red and processed, in less deprived as compared to more deprived older adults. This result is in line with what has already been said, because they are voluptuous foods, rich in fats, not primary necessities, that only people with a specific economic availability can afford to buy. Several studies have demonstrated that the energy density of the diet is inversely linked to the energy cost (37, 38). Foods with a rich content of saturated fats, trans fats and high glycemic index carbohydrates, are cheaper and generally also unhealthy. The intake of this kind of nutrients is associated with increased levels of inflammation that can compromise the state of health (39). A chronic low-grade inflammation typical of abdominal obesity may play a role in the pathogenesis of obesity-related metabolic disorders and metabolic syndrome (40, 41). In fact, high levels of several proinflammatory markers are found in these pathological conditions, including IL-6 (42). In our study, the MD group had higher blood concentrations of IL-6 than the LD group although there were no significant differences between the two groups in terms of BMI and metabolic syndrome. Our results show that MD subjects suffer greater levels of inflammation than LD subjects, despite the prevalent consumption of foods not associated with increased inflammation (43). Further investigation is warranted in this regard. Other studies have already analyzed the association between deprivation, obesity and metabolic syndrome but in a different age population: Sobal and Stunkard describe an association between deprivation and obesity in non-diabetic subjects (44) while La Rosa et al. demonstrated that deprivation is an independent determinant of the metabolic syndrome, which is especially common among elderly deprived women (45). The results of our study on diet and social deprivation could be extended more generally to the older population of Southern Italy and not only to our cohort. In the south, eating habits and socio-economic status are fairly overlapping in this part of the population (24).

### Strengths and Limitations

The present study evaluated the association between diet and social deprivation in older Italian adults. This is its main strength because to our knowledge no study has yet analyzed these aspects in similar populations. Another strength was the identification of a dietary pattern associated with deprivation through a strong machine learning approach. The dietary pattern associated with social deprivation is to be understood as an association ranking of certain food groups with a certain outcome (social deprivation, in this case). However, some limitations must also be taken into account. One of these is the nature of the study which was cross-sectional, and does not allow a clear directionality of an association to be discerned. A further limitation is the FFQ as a dietary assessment method. It is memory-based and thus considered prone to measurement error, limiting the ability to study small diet-disease relationships, in particular (46). In addition, the Dip-Q is a good measure for specific deprivation in different domains (wealth, health, and social engagement) but it is not compatible with other standard deprivation measurements that study geographic areas rather than single individuals (17). Nevertheless, despite the reported limitation of the assessment methods, FFQs remain the dietary assessment method most commonly used to study dietary patterns and population habits (47). Furthermore, a limitation of the machine learning approach is that it cannot explain any biological reasons for the associations detected.

## Conclusion

In conclusion, eating habits, diet quality, and composition are influenced by several factors, including age, socioeconomic, cultural, and educational status. The results of our study are in line with Townsend's idea of social deprivation, in the sense that not only economic but also social, physical, psychological and nutritional conditions influence the state of health. The present results showed that older subjects with a low educational level were more deprived, and ate a diet based on foods that are generally cheaper and more easily available. The dietary pattern associated with social deprivation identified by the current study includes white meat and low-fat dairy products, that were positively associated with deprivation, while wine, leafy vegetables, seafood/shellfish, eggs, dairy, processed and red meat were negatively associated. Reducing the level of deprivation in older subjects could have a positive impact on their eating habits, their dietary quality and hence on their overall state of health during aging.

## Data Availability Statement

The raw data supporting the conclusions of this article will be made available by the authors, without undue reservation.

## Ethics Statement

The studies involving human participants were reviewed and approved by the IRB of IRCCS Saverio de Bellis. The patients/participants provided their written informed consent to participate in this study.

## Author Contributions

LL and RS: conceptualization and methodology. FC, RZ, IB, SD, GC, and DL: investigation. RD: formal analysis. RT and LL: writing—original draft preparation. RS and LV: writing—review and editing. FP, ML, GD, and HB: supervision. RS and GG: project administration. GG: funding acquisition. All authors have read and agreed to the published version of the manuscript.

## Funding

This study was funded by the Italian Ministry of Health with the Ricerca Corrente 2019 Grant. The work reported in this publication was granted by the Italian Ministry of Health, under the Studies on Aging Network, at Italian Research Hospitals (IRCCS).

## Conflict of Interest

The authors declare that the research was conducted in the absence of any commercial or financial relationships that could be construed as a potential conflict of interest.

## Publisher's Note

All claims expressed in this article are solely those of the authors and do not necessarily represent those of their affiliated organizations, or those of the publisher, the editors and the reviewers. Any product that may be evaluated in this article, or claim that may be made by its manufacturer, is not guaranteed or endorsed by the publisher.
