# Corrigendum: Dietary customs and social deprivation in an aging population from Southern Italy: A machine learning approach

**DOI:** 10.3389/fnut.2023.1186961

**Published:** 2023-03-31

**Authors:** Rossella Tatoli, Luisa Lampignano, Rossella Donghia, Fabio Castellana, Roberta Zupo, Ilaria Bortone, Sara De Nucci, Giuseppe Campanile, Domenico Lofù, Luigi Vimercati, Madia Lozupone, Giovanni De Pergola, Francesco Panza, Gianluigi Giannelli, Tommaso Di Noia, Heiner Boeing, Rodolfo Sardone

**Affiliations:** ^1^National Institute of Gastroenterology—IRCCS “Saverio de Bellis”, Castellana Grotte, Italy; ^2^Department of Electrical and Information Engineering, Polytechnic of Bari, Bari, Italy; ^3^Interdisciplinary Department of Medicine, Section of Occupational Medicine B. Ramazzini, School of Medicine, University of Bari Aldo Moro, Bari, Italy; ^4^Department of Basic Medicine, Neuroscience, and Sense Organs, University of Bari Aldo Moro, Bari, Italy; ^5^Unit of Internal Medicine and Geriatrics, National Institute of Gastroenterology “S. de Bellis” Research Hospital, Castellana Grotte, Italy; ^6^Department of Biomedical Science and Human Oncology, School of Medicine, University of Bari Aldo Moro, Bari, Italy; ^7^Department of Epidemiology, German Institute of Human Nutrition Potsdam-Rehbruecke, Nuthetal, Germany

**Keywords:** social deprivation, older, diet, population study, dietary pattern

In the published article, there was an error in affiliation 1. Instead of “Unit of Digital Health and Health Technology Assessment for “Salus in Apulia Study,” National Institute of Gastroenterology, “S. de Bellis” Research Hospital, Castellana Grotte, Italy”, it should be “National Institute of Gastroenterology—IRCCS “Saverio de Bellis”, Castellana Grotte, Italy”.

The authors apologize for this error and state that this does not change the scientific conclusions of the article in any way. The original article has been updated.

