# Supplementary material for: Dietary Customs and Social Deprivation in an Aging Population From Southern Italy: A Machine Learning Approach
Source: Front Nutr. 2022 Mar 7;9:811076. doi: 10.3389/fnut.2022.811076 (PMC8942783; doi:10.3389/fnut.2022.811076)
Supplement: Supplementary file 1 [file Table_1.DOCX]

**Supplementary Table 1** Importance scores computed from the RF for each variable for patients with and without social deprivation

| Food-Groups | **Full Model RF** | |  |
| --- | --- | --- | --- |
|  | OOB error ^*^ | Importance Score | |
|  |  |  | |
| Juices | 0.466 | 0.326 | |
| Beer | 0.462 | 0.336 | |
| Spirits | 0.452 | 0.373 | |
| Coffee | 0.460 | 0.417 | |
| Caloric Drinks | 0.460 | 0.439 | |
| Sweets | 0.464 | 0.448 | |
| Legumes | 0.488 | 0.467 | |
| Grains | 0.478 | 0.470 | |
| Root Vegetables | 0.478 | 0.482 | |
| Sugary foods | 0.462 | 0.483 | |
| Olive/Veg.Oil | 0.464 | 0.483 | |
| Fruits | 0.478 | 0.497 | |
| Ready to Eat Dish | 0.468 | 0.497 | |
| Potatoes | 0.470 | 0.503 | |
| Nuts | 0.474 | 0.503 | |
| Water | 0.468 | 0.507 | |
| Fruiting Vegetables | 0.462 | 0.518 | |
| Other Vegetables | 0.480 | 0.519 | |
| Fish | 0.446 | 0.526 | |
| Wine | 0.472 | 0.538 | |
| Leafy Vegetables | 0.464 | 0.552 | |
| Seafood/Shellfish | 0.472 | 0.565 | |
| Processed Meat | 0.460 | 0.616 | |
| Red Meat | 0.472 | 0.683 | |
| White Meat | 0.454 | 0.735 | |
| Low-Fat Dairy | 0.450 | 0.812 | |
| Eggs | 0.436 | 0.826 | |
| Dairy | 0.449 | 1.000 | |

^*^ Error rate and stability of results of backwards elimination of variables using OOB error, evaluated using 500 boostrap samples.

**REFERENCES**

1. Genuer R, Poggi J-M, Tuleau-Malot C. Variable selection using Random Forests. Pattern

Recognition Letters 2010;31(14):2225-2236.

2. Behnamian A, Millard K, Banks SN, White L, Richardson M, Pasher J. A systematic approach

for variable selection with random forests: Achieving stable variable importance values. IEEE

Geoscience and Remote Sensing Letters 2017;14(11):1988-1992.
